# *In silico* characterization of the interaction of AKR1B1 with the deacetylase activation domain (DAD)

**DOI:** 10.3389/fmolb.2025.1562206

**Published:** 2025-04-16

**Authors:** Francisco J. Gómez-Zaldívar, Luis Córdova-Bahena, Angel J. Ruiz-Moreno, Paolo Ceppi, Marco A. Velasco-Velázquez

**Affiliations:** ^1^ School of Medicine, Universidad Nacional Autónoma de México (UNAM), Mexico City, Mexico; ^2^ Graduate Program in Biochemical Sciences, Universidad Nacional Autónoma de México, Mexico City, Mexico; ^3^ Investigadores por México, Secretaría de Ciencia, Humanidades, Tecnología e Innovación (SECIHTI), Mexico City, Mexico; ^4^ Interdisciplinary Center for Clinical Research (IZKF), Friedrich-Alexander Universität Erlangen-Nürnberg, Erlangen, Germany; ^5^ Department of Biochemistry and Molecular Biology, University of Southern Denmark, Odense, Denmark

**Keywords:** AKR1B1, deacetylase activation domain, nuclear receptor corepressor, molecular dynamics, binding sites prediction, protein-protein interaction, in silico mutagenesis

## Abstract

The aldo-keto reductase family 1 member B1 (AKR1B1) plays a key role in cancer progression by competing with histone deacetylase 3 to bind to the deacetylase activation domain (DAD) of the nuclear receptor corepressor SMRT. Previous studies showed that the L289A mutation in AKR1B1 disrupts its ability to form a dimer with DAD but further details of this interaction remain uncharacterized. This study aimed to model the DAD/AKR1B1 dimer by molecular docking and characterize the complex using molecular dynamics simulations. We identified a new model with increased structural stability for AKR1B1, reduced disruption of secondary structures of DAD, and lower ΔG than a previously reported one. *In silico* mutagenesis of AKR1B1 assessed the contributions from individual residues. We identified six hotspot residues that mediate the complex interface. Those residues are located in the α8 and H2 alpha helices of AKR1B1 and include the experimentally determined L289. These results propose new hypotheses regarding the interaction between DAD and AKR1B1, guiding future experimental approaches.

## 1 Introduction

The human aldo-keto reductase family 1 member B1 (AKR1B1) is a multifunctional enzyme involved in diverse biological processes. The physiological role of AKR1B1 primarily involves the conversion of glucose to sorbitol in the first reaction of the polyol pathway. However, AKR1B1 also participates in detoxification, steroid metabolism, and the oxidative stress response (Zhao et al., 2024). AKR1B1 is considered a pharmacological target due to its role on diabetic inflammatory pathologies, overexpression in various types of cancer, and its role in promoting epithelial-mesenchymal transition (Banerjee and Turksen, 2021; Schwab et al., 2018). For example, silencing AKR1B1 expression in triple-negative breast and non-small cell lung cancer cells significantly decreases cell migration, growth rate, and colony formation (Schwab et al., 2018; Khayami et al., 2020). AKR1B1 also plays a role in the physiopathology of diabetic nephropathy and retinopathy, and of some inflammatory disorders. Consequently, multiple inhibitors of AKR1B1 enzymatic activity have been developed (Balestri et al., 2022; Liu et al., 2024). However, the use of inhibitors of the enzymatic activity of AKR1B1 fails to phenocopy the effects of *AKR1B1* genetic silencing (Schwab et al., 2018). Therefore, it has been hypothesized that the role of AKR1B1 in maintaining an aggressive phenotype in cancer cells may be mediated by catalytic-independent mechanisms (Banerjee and Turksen, 2021).

Thiagarajan et al. (Thiagarajan et al., 2016) reported that AKR1B1 forms protein-protein interactions (PPIs), competing with histone deacetylase 3 (HDAC3) to bind the deacetylase activation domain (DAD) present in the nuclear receptor corepressors 1 (NCOR1) and 2 (NCOR2, also known as Silencing Mediator of Retinoic acid and Thyroid hormone receptor -SMRT-). The region of HDAC3 mediating its interaction with DAD is distinct from its catalytic site (Supplementary Figure S1). Thus, it is possible that the pro-oncogenic activities of HDAC3 (Tong et al., 2020) and NCOR1/2 (Battaglia et al., 2010) could be regulated by AKR1B1-DAD binding.

The reported AKR1B1-DAD interaction occurs outside the AKR1B catalytic site. Increasing concentrations of recombinant DAD promote the formation of complexes in co-immunoprecipitation assays but have a mild effect in the enzymatic activity of ARK1B1 (Thiagarajan et al., 2016). Site-directed mutagenesis assays identified L289 of AKR1B1 as a key residue for maintaining the AKR1B1/DAD complex (Thiagarajan et al., 2016). However, the structural details of this interaction remain unclear. Further analysis of the interaction of DAD with AKR1B1 will open new opportunities for the modulation of the protumoral activities AK1RB1 and NCOR1/NCOR2. Thus, we used multiple *in silico* approaches for defining and characterizing the DAD binding site on AKR1B1. We aimed to generate new hypotheses about the structural determinants of this interaction, which could guide future experiments.

## 2 Methodology

### 2.1 Protein structure retrieval

The structures of human AKR1B1 and HDAC3/DAD complex were obtained from Protein Data Bank (PDB) IDs 6F7R (Castellví et al., 2019), and 4A69 (Watson et al., 2012), respectively. Human AKR1B1 structure was chosen from a collection of 125 available crystallographic structures that included co-crystallized inhibitors and the nicotinamide adenine dinucleotide phosphate (NADP^+^) cofactor. To ensure structural consistency, all structures were aligned using mTM-align (Dong et al., 2018) and clustered. The structure from the largest cluster with the best crystallographic resolution (0.92 Å) was selected as the representative model.

### 2.2 Molecular docking

The protein-protein molecular docking was performed by using three different protocols: PatchDock (Schneidman-Duhovny et al., 2005), ZDOCK (Pierce et al., 2011), and HADDOCK (Dominguez et al., 2003). PatchDock utilized a geometry-based algorithm that evaluated shape complementarity between proteins, combined with the Random Sample Consensus search algorithm. ZDOCK employed a Fast Fourier Transform-based method, incorporating electrostatic and desolvation energy terms, with a grid resolution of 1.2 Å. HADDOCK, a flexible docking approach, was applied using rigid-body docking, followed by refinement through simulated annealing in torsional space to optimize the binding interface. To improve the sample efficiency, all ligands, ions, and water molecules of the proteins were removed before docking. However, the AKR1B1 cofactor NADP+ was retained because part of it remains exposed to the solvent and could influence PPIs.

The top 30 solutions from each docking server were analyzed by hierarchical clustering to identify common binding modes, reducing redundancy among the docking poses. Within the largest cluster, the top-ranked complexes from each server were identified and their ΔG at 25°C were calculated using the protein binding energy prediction (PRODIGY) web server (Xue et al., 2016). The solution with the lower ΔG was selected as the best model for further analyses. When using ZDOCK or HADDOCK docking servers, L289 of AKR1B1 was selected as a hotspot, integrating prior knowledge (Thiagarajan et al., 2016). Finally, the interface between AKR1B1 and DAD was analyzed using PDBePISA (Krissinel and Henrick, 2007) and the residues involved in PPIs were defined by using PyMol (Schrödinger and DeLano, 2020).

### 2.3 Molecular dynamics (MD) simulations

Two AKR1B1/DAD protein-protein complexes were compared in MD studies. The first complex was obtained by aligning residues 266-312 of AKR1B1 with residues 9-49 of HDAC3, as previously reported (Thiagarajan et al., 2016). Those regions contain a motif with sequence similarity and structural identity (Thiagarajan et al., 2016). The second complex was generated through molecular docking as described above. As a control, unbound AKR1B1 was evaluated. All-atom MD simulations were performed in GROMACS (Abraham et al., 2015) using the force field CHARMM36m (Huang et al., 2017). All systems were prepared by using the CHARMM-GUI (Jo et al., 2017) server. First, each protein-protein complex or the uncomplexed AKR1B1 was placed within a cubic box having an edge length of 78 Å. Each box was fully solvated using the TIP3P model for water molecules, and K^+^ and Cl^−^ ions were added to neutralize the charge and achieve an ionic concentration of 0.15 M. Second, energy minimization was carried out for 100000 steps employing the steepest descent algorithm. Subsequently, equilibration was conducted under an NVT and NPT ensembles for 500 ps each at 310.15 K and 1 atm, employing V-rescale thermostat and Berendsen barostat, respectively. The LINCS algorithm (Hess et al., 1997) was applied to all bonds involving hydrogen atoms. Finally, MD simulations were generated for 500 ns with an integration time frame of 2 fs. The Root Mean Square Deviation (RMSD), Root Mean Square Fluctuation (RMSF), Radius of gyration (R_g_), and helicity were calculated by using MDAnalysis (Gowers et al., 2016), and the trajectories were visualized with VMD (Humphrey et al., 1996). Intermolecular interactions analysis was carried out by a python tailored-made script implementing MDAnalysis.

The ΔG was calculated using the gmx_mm/pbsa tool (Kumari et al., 2014), employing the entire trajectories from the MD simulations with intervals of 0.1 ns. Additionally, per-residue energy decomposition of the ΔG calculations using the MM/PBSA approach were conducted to identify key residues involved in PPI, considering van der Waals, electrostatic, and solvation contributions.

### 2.4 *In silico* alanine mutagenesis

We generated seven single AKR1B1 mutants (AKR1B1mut) to assess the influence of specific residues on PPI. The residues that were identified in per-residue energy decomposition as significant contributors to PPI were selected to generate the following single AKR1B1mut: E193A, Q197A, S282A, T286A, L289A, and S290A. Mutants were generated *in silico* by substituting the target residues with alanine. MD simulations were conducted for the AKR1B1mut/DAD systems and ΔG was calculated following the aforementioned protocols. Simulations were carried out as single replicates; thus, results should be interpreted accordingly. Additionally, a second alanine scanning mutagenesis was performed by submitting the AKR1B1/DAD complex structure (PDB format) to the DrugScorePPI server (Krüger and Gohlke, 2010). The analysis was conducted using default parameters to calculate the per-residue ΔG contribution upon alanine substitution.

## 3 Results

### 3.1 Modeling of the binding mode for DAD on AKR1B1

We first aimed to identify a proper docking protocol for modeling the binding mode of AKR1B1 to DAD. Thus, we analyzed the ability of three distinct protein-protein molecular docking protocols to accurately reproduce the reported crystal binding mode of DAD to HDAC3. Hierarchical clustering of 90 solutions obtained from three docking servers revealed that 11 solutions and the reported crystallographic conformation clustered together (Supplementary Figure S2A). Notably, the best-ranked solution from each protocol was encompassed within that cluster. The RMSD comparing the best-ranked binding modes from each protocol ranged from 0.97 to 1.82 Å (Supplementary Figures S2B, C), indicating that the strategy of using three different docking protocols and analyzing them by hierarchical clustering effectively replicated the binding mode of DAD in HDAC3 as identified by x-ray crystallography.

**FIGURE 1:**
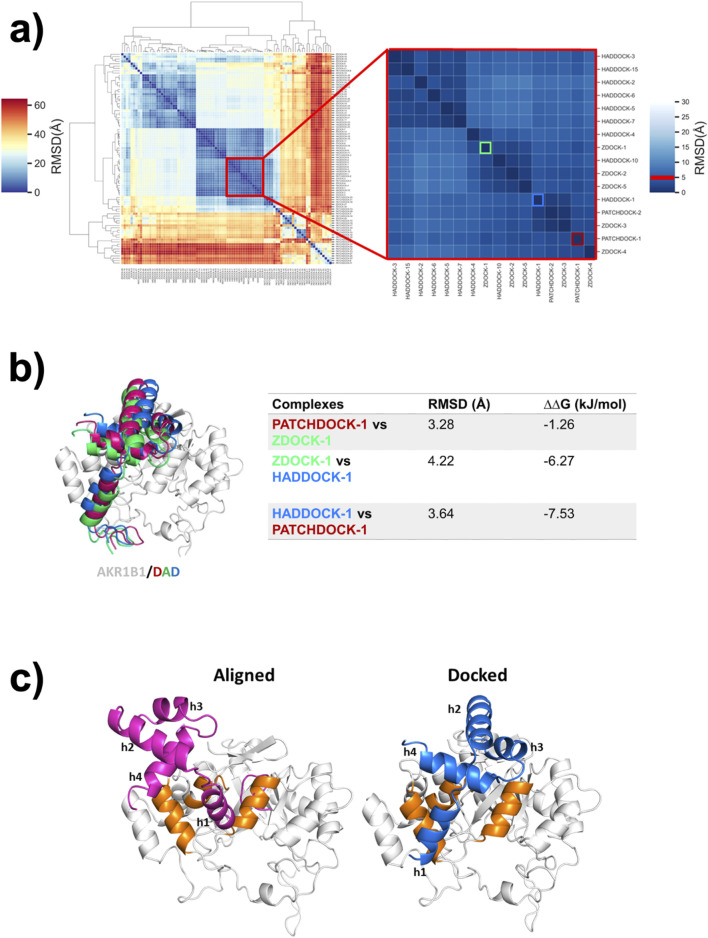
A new binding model for the DAD/AKR1B1 complex. **(a)** Hierarchical clustering of conformations obtained by DAD docking on AKR1B1. Inset shows the largest subcluster, containing 16 poses with RMSD ≤5 Å. Conformations are labeled using the docking server names and a number indicating the ranked position. The best-scoring conformation calculated by PatchDock, ZDOCK, and HADDOCK are framed on red, green, and blue respectively. **(b)** Comparison of the best-ranked poses identified in **(a)**. The RMSD between DAD conformations obtained from each server and the differences in the free Gibbs energy of the complexes are shown in the table. **(c)** DAD/AKR1B1 complexes reported by Thiagarajan et al. (2016) (aligned) and the best-ranked solution of HADDOCK (docked). DAD is colored in pink in the aligned model and blue in the docked one. AKR1B1 alpha helices participating in binding to DAD are highlighted in orange.

The same strategy was then employed to generate a new model for the binding mode of DAD to AKR1B1. Hierarchical clustering of 90 solutions obtained from docking revealed that the largest subcluster of AKR1B1/DAD models comprised 16 solutions, including the best-ranked solutions from each of the three protocols (Figure 1a). However, comparison of the best-ranked solutions showed that the RMSD for the alpha carbons of DAD ranged from 3.28 to 4.22 Å (Figure 1b). Interestingly, the buried surface area varied across the different predictions. The interface areas of the AKR1B1/DAD complexes generated with PatchDock, HADDOCK, and ZDOCK were 748.5, 913.6, and 980.3 Å^2^, respectively. In order to select a model for further work, we predicted the ΔG of the three best-ranked poses using PRODIGY (Xue et al., 2016). Despite using the same model to calculate the ΔG, significant differences were observed between the best-ranked poses obtained with the employed docking algorithms. The binding mode identified by HADDOCK showed lower ΔG (Figure 1b) and was therefore selected for further analysis.

Another DAD/AKR1B1 binding mode, generated by alignment, was reported by Thiagarajan et al. (2016). Comparison between our docking-derived model and the previously reported aligned model revealed important differences in DAD orientations. The helices of DAD were labeled as h1, h2, h3, and h4. In both models, h2 remains exposed to the solvent while h1 and h4 play major but different roles in each binding mode. In the aligned complex, h1 primarily remains buried within AKR1B1 and h4 has limited involvement in the binding. In contrast, in the docked complex both h1 and h4 directly face AKR1B1 in a balanced manner. Furthermore, h3 is oriented directly toward AKR1B1 in the docked complex, participating in the PPI, whereas its role is negligible in the aligned complex (Figure 1c).

### 3.2 DAD/AKR1B1 model obtained by molecular docking outperformed a previous model

**FIGURE 2:**
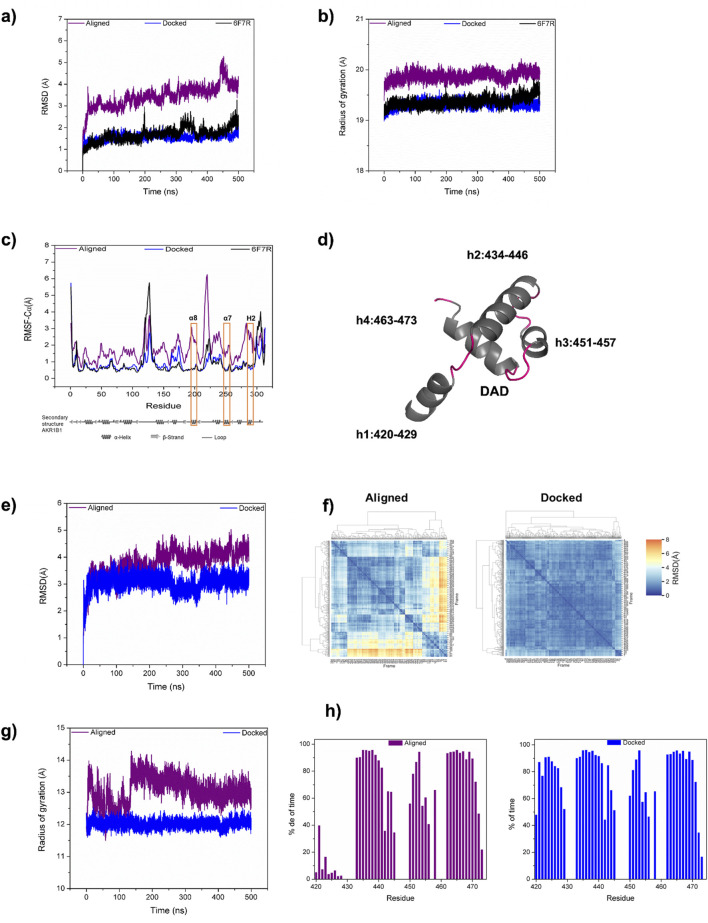
Comparison of DAD/AKR1B1 model identified by docking with the previously reported model. **(a–c)** Backbone RMSD **(a)**, radius of gyration **(b)**, and alpha carbon RMSF **(c)** for AKR1B1 in aligned (purple) and docked (blue) complexes, obtained from 500 ns of MD simulation. For comparison the unbound AKR1B1 (PDB code 6F7R) is presented (black). Orange rectangles in **(c)** label the AKR1B1 alpha helices α8, α7, and H2, which participate in binding. **(d)** Structural representation for the four DAD helices. **(e, f)** Backbone RMSD of DAD during MD evolution **(e)**, and between 500 DAD conformational states **(f)**. **(g, h)** Radius of gyration **(g)** and helicity **(h)** for both aligned and docked DAD.

To further analyze the DAD/AKR1B1 complexes generated by alignment and by docking, we performed MD studies of both models. As a control, we performed MD simulation for the uncomplexed AKR1B1 using the structure PDB ID 6F7R. As was expected, AKR1B1 showed minimal conformational changes in absence of DAD, exhibiting an RMSD of 1.68 (±0.31) Å. Within the docked complex, AKR1B1 displayed equivalent stability with an RMSD of 1.54 (±0.14) Å, whereas in the aligned complex, the protein exhibited an RMSD of 3.44 (±0.51) Å (Figure 2a), suggesting that the starting binding mode is unstable.

Computation of the radius of gyration (R_g_) showed that the uncomplexed protein exhibits a mean R_g_ of 19.37 (±0.09) Å, indicating inherent compactness and stability in its structure. The mean R_g_ of 19.32 (±0.07) Å in the docked complex suggested that the PPI between AKR1B1 and DAD does not compromise AKR1B1 stability. Conversely, the R_g_ values for AKR1B1 in the aligned complex (mean R_g_ 19.87 ±0.09 Å) were consistently higher to those observed in the docked complex (Figure 2b), indicating a more diffuse conformation in the aligned complex, which may reduce protein stability.

We also computed the RMSF of the systems to analyze residue flexibility. The AKR1B1 DAD-binding domain comprises three helices: α8, α7, and H2. The uncomplexed protein displayed high fluctuation (2.5 Å) for a residue in the interconnection loop between α8 and α7 helices. In comparison, such fluctuation decreased by approximately 1 Å in the docked complex due to the role of that loop in the binding behaviour. Conversely, a fourfold increase in fluctuation was observed for that loop in the aligned complex. Similarly, the α7 and H2 interconnection loop of AKR1B1 showed higher fluctuation in the aligned complex compared to both the docked complex and the unbound protein. The lower fluctuation of AKR1B1 in the docked complex demonstrates a more stable binding behavior compared to the aligned complex (Figure 2c).

We also analyzed the stability of DAD (Figure 2d) in our MD simulations. The RMSD for DAD in the docked and aligned complexes were 3.05 (±0.28) Å and 3.79 (±0.48) Å, respectively. The reduced mean RMSD and standard deviation in the docked complex indicate lower displacement from the initial conformation and fewer conformational changes for DAD compared to the aligned one (Figure 2e). Moreover, hierarchical clustering of 500 conformational states, sampled every 1 ns during MD simulations, revealed increased stability in the docked protein compared to the aligned (Figure 2f). Additionally, the R_g_ of DAD were 12.02 (±0.11) Å for the docked complex and 13.05 (±0.41) Å for the aligned complex, indicating a more compact conformation and corroborating the stability of our docked model (Figure 2g). Furthermore, the helicity of DAD remained stable in the docked complex, but in the aligned complex the helicity of h1 persisted for less than 40% of the simulation time, weakening its ability to mediate AKR1B1 binding (Figure 2h). Finally, the ΔG for the complex generated by docking was −172.00 ± 2.37 kJ/mol whereas for the aligned complex it was −18.83 ± 3.22 kJ/mol, indicating that the docked complex was energetically more favorable than the aligned complex. Altogether, the comparison of the dynamic binding behavior of two DAD/AKR1B1 binding models indicate that the new one proposed here has advantages over the one previously published by Thiagarajan et al. (2016).

### 3.3 Definition of DAD-binding interface on AKR1B1

**FIGURE 3:**
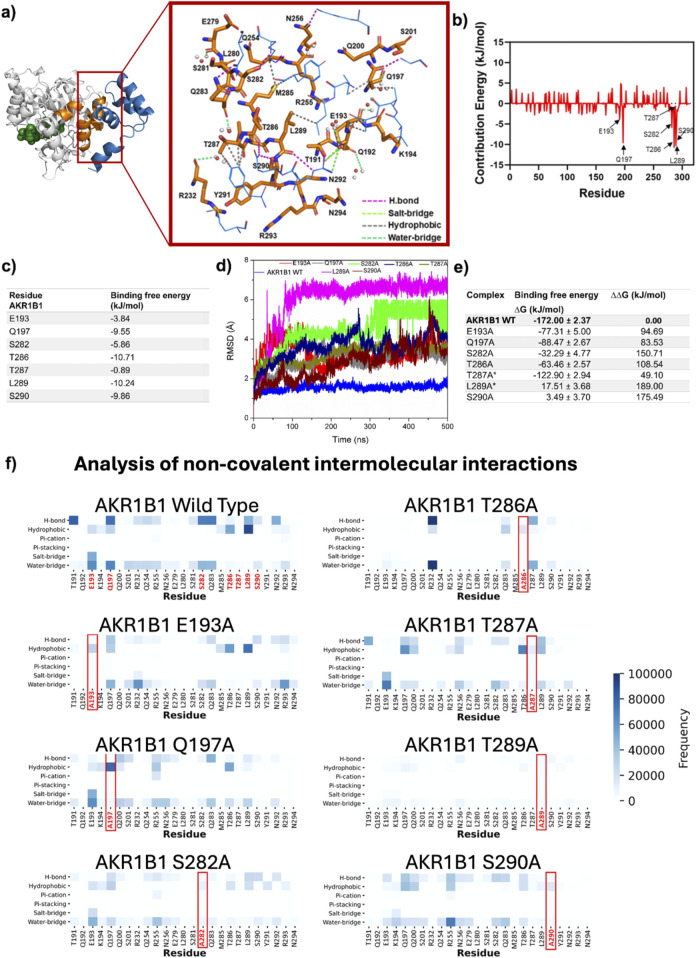
Relevance of the residues mediating DAD binding to AKR1B1. **(a)** Representative intermolecular interactions on DAD-AKR1B1 interface. AKR1B1 residues are colored in orange and shown on licorice model whereas DAD residues are colored in blue and shown on wire model. NADP+ is displayed as green spheres. **(b,c)** Energy contribution by residue for all AKR1B1 residues in the interface **(b)** and for the seven main contributors **(c)**. **(d)** Backbone RMSD from MD simulations with seven single mutants. **(e)** ΔG after mutation and differences between mutated and wild type complexes. The asterisk indicates that the mutation has been validated as important for binding by *in vitro* assays (Thiagarajan et al., 2016). **(f)** Non-covalent interactions quantification for the complete set of conformational states accessible during MD simulation for complexes containing wild type and mutant AKR1B1.

The most prevalent intermolecular interactions at the AKR1B1/DAD interface were identified by analyzing the dynamic behavior of the newly proposed binding mode. In AKR1B1, L289 exhibited the highest number of hydrophobic interactions. Additionally, high prevalence of hydrogen bonds was observed with residues T191, Q197, S282, Q283, L289, and N292, whereas a salt bridge with the negative charged residue E193 was recurrently formed (Figure 3a).

Analysis of the per-residue contribution to the ΔG highlighted the significant roles of AKR1B1 residues T286 and L289, which displayed energetic contributions <-10 kJ/mol. Additionally, AKR1B1 residues S290 and S282, located in the H2 helix, and Q197 and E193, found in the α7 helix, showed important energetic contributions, ranging from −3.84 to −9.86 kJ/mol (Figures 3b,c). Residues in the α8 helix displayed negligible involvement in the PPI.

The role of each of the six residues identified as relevant was defined by generating AKR1B1 single alanine mutants and analyzing their DAD-binding behavior in MD simulations. A seventh mutant, T287A, was included in our analyses as control since its role in binding was previously described as irrelevant by site-directed mutagenesis (Thiagarajan et al., 2016). The mean RMSD for alpha carbons in the different AKR1B1mut ranged from 2.76 ±0.83 (S290A) to 6.02 ±1.29 Å (L289A), contrasting with the 1.55 ±0.14 Å observed in the wild type (WT) protein (Figure 3d). Therefore, we observed significant conformational rearrangements in all AKR1B1mut. Computation of ΔG for the complexes showed that AKR1B1mut L289A and S290A had unstable binding, as indicated by the positive value of ΔG. In addition, ΔΔG, calculated as the difference between ΔG of the complex containing AKR1B1 wild type and those with AKR1B1mut, revealed destabilizing effects across all mutants (Figure 3e). Altogether, these results suggest that single mutations on the proposed DAD-binding domain of AKR1B1 destabilize the PPI.

Analysis of intermolecular interactions revealed specific effects of each mutation. L289A mutation generated disruptions to the majority of interactions mediated in all the interface residues. Similarly, S290A mutation resulted in the disappearance of hydrogen bonds and decreased interactions by other residues, except for R255 in α7, which formed several water bridges. S282A and Q197A mutants were unable to form the hydrogen bonds present in the WT protein and switched to hydrophobic interactions. The T286A mutant displayed decreased hydrophobic interactions and the emergence of hydrogen bonds and water bridges with R232 in α7. The E193A mutation generated a loss of salt bridges and water bridges, while hydrophobic interactions remained. Finally, T287A showed minimal impact on the interaction profile (Figure 3f). Interestingly, alanine scanning of the binding model reported here revealed that residues Y291 and E279, flanking the DAD-contact region on AKR1B1 H2 helix, displayed ΔΔG >2.5 KJ/mol (Supplementary Table S1), suggesting that additional residues may also play a role in the PPI. Further studies will be necessary to explore their role in detail.

## 4 Discussion and conclusion

**FIGURE 4:**
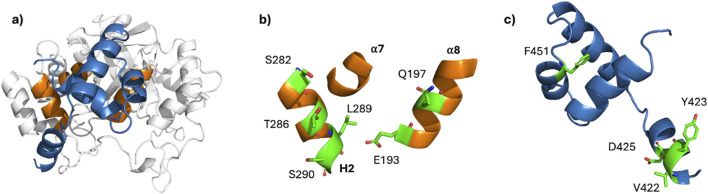
Binding domains in AKR1B1 and DAD. **(a)** Representative conformation of the DAD/AKR1B1 complex. **(b, c)** Residues identified as principal contributors to ΔG for AKR1B1 **(b)** and DAD **(c)**. The key residues are shown in green with their lateral chains as sticks.

Utilizing structural data and protein-protein molecular docking, we proposed a new model of DAD/AKR1B1 complex (Figure 4a) and characterized its dynamic behavior. When compared to a model previously reported by Thiagarajan et al. (2016), our model: i) showed a new orientation of DAD; ii) displayed increased structural stability for AKR1B1; had reduced disruption of secondary structures of DAD; and iv) exhibited significantly lower ΔG.

Our results demonstrate that the conformation of HDAC3-bound DAD (Watson et al., 2012) is functional for binding to AKR1B1, implying that such conformation can be targeted by at least two different partner proteins. The ability of DAD to form complexes with different partners partially explains the multiple functions identified for DAD (Li et al., 2021). DAD binding to HDAC3 promotes the enzymatic activity of the latter (Emmett and Lazar, 2019). In this way, NCOR1 recruits HDAC3 to drive leukemogenesis by transcriptional repression of myeloid-differentiation genes (Wan et al., 2019), and promotes proliferation and a proinflammatory senescence-associated secretory phenotype in colon cancer cells (St-Jean et al., 2021). In contrast, the enzymatic activity of AKR1B1 is only slightly affected by DAD binding (Thiagarajan et al., 2016), suggesting that DAD-AKR1B1 interaction prevents their binding to other effector proteins.

Exhaustive analysis of the role of AKR1B1 residues at the DAD-binding site identified six AKR1B1 residues as the principal contributors to ΔG (Figure 4b), and their counterpart in DAD (Figure 4c). Noteworthy, we found a pivotal role of leucine 289 and a negligible participation of threonine 287, which is consistent with the experimental evidence previously reported (Thiagarajan et al., 2016). Furthermore, *in silico* mutation of serine 290 had an important impact on the stability of the complex. So, our results propose serine 290 as a critical residue for AKR1B1-DAD and should be studied in site-directed mutagenesis experiments.

In summary, the new model proposed here and its comprehensive characterization establish new hypotheses about the way DAD and AKR1B1 interact, directing future experiments. If our predictions are confirmed, they could contribute to the development of strategies aimed at modulating the catalytic-independent protumoral functions of AKR1B1.

## Data Availability

The raw data supporting the conclusions of this article will be made available by the authors, without undue reservation. The top-ranked AKR1B1/DAD complexes obtained by docking are available at https://doi.org/10.6084/m9.figshare.28548674.v1.
