# Supplementary material for: In silico characterization of the interaction of AKR1B1 with the deacetylase activation domain (DAD)
Source: Front Mol Biosci. 2025 Apr 16;12:1562206. doi: 10.3389/fmolb.2025.1562206 (PMC12040874; doi:10.3389/fmolb.2025.1562206)
Supplement: Supplementary file 1 [file DataSheet1.pdf]

In silico characterization of the interaction of AKR1B1 with deacetylase activation domain (DAD)

Francisco Gómez-Zaldívar, Luis Córdova-Bahena, Angel Ruiz-Moreno, Paolo Ceppi, Marco A. Velasco-Velázquez

Supplementary Material

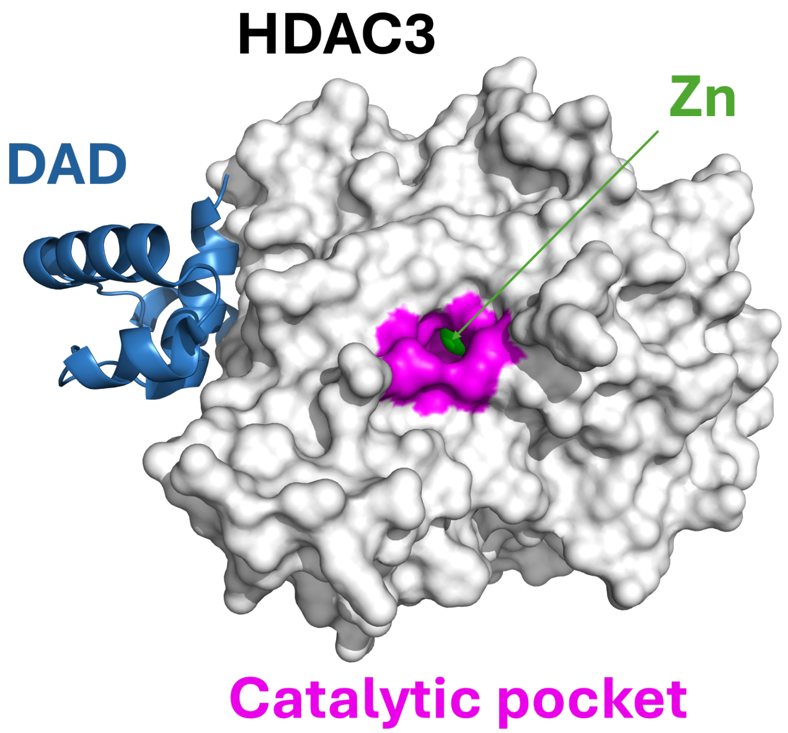

Supplementary Figure 1. **HDAC3-DAD complex**. Molecular surface representation of HDAC3 bound to DAD (blue cartoon) generated using the crystallographic structure with PDB ID 4A69. The catalytic pocket of HDAC3 is highlighted in magenta, with the zinc cofactor shown as a green sphere.

a)

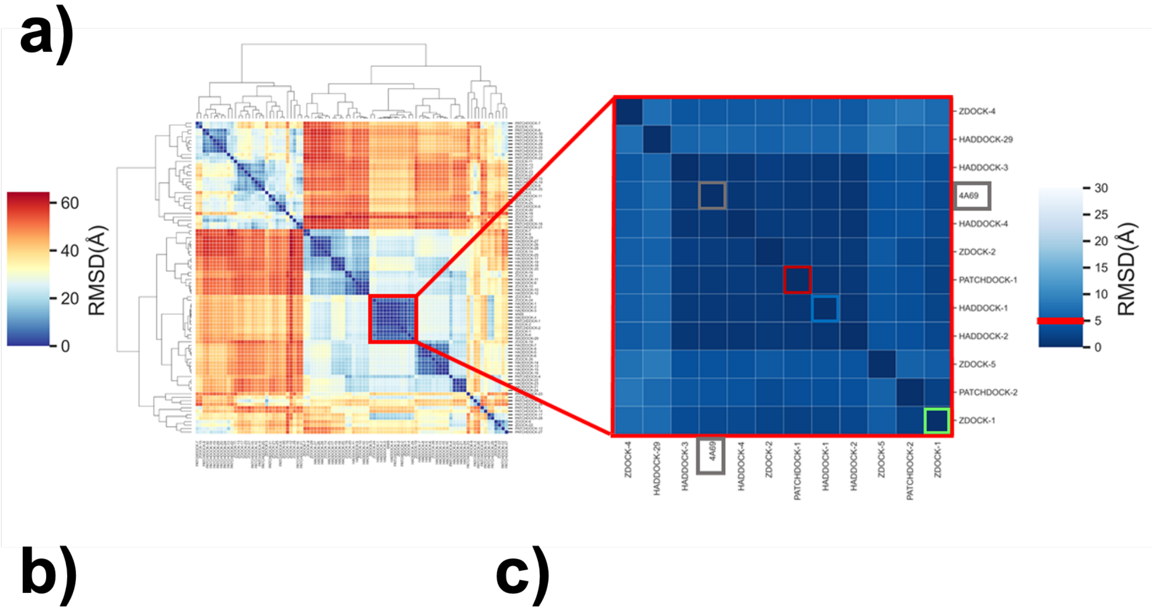

b)

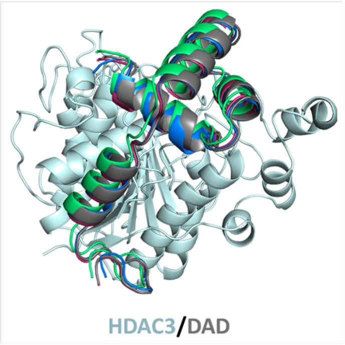

c)

Complex	RMSD (Å) vs 4A69
PatchDock-1	1.28
ZDOCK-1	1.82
HADDOCK-1	0.97

Supplementary Figure 2. Docking protocol reproduces the binding mode for DAD/HDAC3 complex. (a) Hierarchical clustering of 91 conformations for DAD on HDAC3. Inset shows the largest subcluster with 12 conformations for HDAC3. DAD conformations are labeled using docking server names and a number indicating the ranked position. The crystallographic conformation of DAD on HDAC3 is labeled as PDB code 4A69 and highlighted in gray. (b) The best-scoring conformation calculated by PatchDock, ZDOCK, and HADDOCK servers are colored on red, green, and blue respectively. (c) RMSD between DAD conformations are reported.

Table S1. Alanine scanning for the newly identified binding model of AKR1B1/DAD.

AKR1B1 residue	ΔΔG, kJ/mol	AKR1B1 residue	ΔΔG, kJ/mol
T191A	1.21	L280A	0.88
**E193A**	0.92	S281A	0.96
K194A	0.50	**S282A**	2.93
I196A	0.96	Q283A	-0.29
**Q197A**	7.70	M285A	1.17
Y198A	0.50	**T286A**	1.17
Q200A	0.25	**T287A**	1.26
S201A	0.54	L288A	0.33
R232A	0.71	**L289A**	1.97
Q254A	-0.71	**S290A**	1.05
R25A	1.46	Y291A	2.51
N256A	0.64	N292A	1.38
E279A	2.72		

Mutations analyzed by MD simulations are shown in bold.
